# Structure of a Sialo-Oligosaccharide from Glycophorin in Carp Red Blood Cell Membranes

**DOI:** 10.3390/membranes4040764

**Published:** 2014-11-13

**Authors:** Takahiko Aoki, Kenji Chimura, Hikaru Sugiura, Yasuko Mizuno

**Affiliations:** 1 Laboratory of Quality in Marine Products, Graduate School of Bioresources, Mie University, 1577 Kurima Machiya-cho, Tsu, Mie 514-8507, Japan; E-Mails: c.blacky@softbank.ne.jp (K.C.); mie_uni1423@me.com (H.S.); 2 Toray Research Centre, Inc., 1111 Tebiro, Kamakura, Kanagawa 248-8555, Japan; E-Mail: Yasuko_Mizuno@trc.toray.co.jp

**Keywords:** fish, red blood cell membranes, erythrocyte, glycophorin, oligosaccharide, sialic acid, *N*-glycolylneuraminic acid

## Abstract

We isolated a high-purity carp glycophorin from carp erythrocyte membranes and prepared the oligosaccharide fraction from glycophorin by β-elimination [1]. The oligosaccharide fraction was separated into two components (P-1 and P-2) using a Glyco-Pak DEAE column. These O-linked oligosaccharides (P-1 and P-2) were composed of glucose, galactose, fucose, *N*-acetylgalactosamine and *N-*glycolylneuraminic acid (NeuGc). The P-1 and P-2 contained one and two NeuGc residues, respectively, and the P-1 exhibited bacteriostatic activity [1]. Using NMR and GC-MS, we determined that the structure of the bacteriostatic P-1 was NeuGcα2→6 (Fucα1→4) (Glcα1→3) Galβ1→4GalNAc-ol. This O-linked oligosaccharide was unique for a vertebrate with respect to the hexosamine and hexose linkages and its non-chain structure.

## 1. Introduction

Glycophorins and band-3 proteins found in mammalian [2,3] and avian [4,5,6,7] erythrocyte membranes are transmembrane glycoproteins. Glycophorins are members of the sialoglycoprotein family and contain more sialic acid than band-3 proteins [8]. The glycophorins function as MN blood group antigens [9] or virus receptors [10,11]. Despite the importance of glycophorins to cell function and immunity, little is known about their function in teleost blood cells.

In a previous paper, we determined the sialic acid content as *N-*glycolylneuraminic acid (NeuGc) and the chemical composition of glycophorin isolated from carp erythrocyte membranes [1]. The O-linked sialo-oligosaccharides were prepared from carp glycophorin by β-elimination, and the oligosaccharide fraction was separated into two components (P-1 and P-2) using a Glyco-Pak DEAE column. The P-1 and P-2 components contained one and two NeuGc residues, respectively. The P-1 exhibited bacteriostatic activity and the P-2 fraction, which lacked sialic acid, exhibited no bacteriostatic sensitivity [1].

The objective of this study was to determine the structure of this monosialyl oligosaccharide (P-1) from carp glycophorin.

## 2. Materials and Methods

### 2.1. Materials

Live carp (*Cyprinus carpio*) were obtained from a local fish market. *N-*glycolylneuraminic acid (NeuGc) was obtained from Sigma Chemical Co. (St. Louis, MA, USA). The GL-Pak Carbograph cartridge (500 mg/6 mL) was purchased from GL Sciences Inc. (Tokyo, Japan). All other reagents were analytical grade. 

### 2.2. Preparation and Isolation of Glycophorin from the Red Blood Cell Membranes

The erythrocyte membranes were prepared according to our previous study [1]. The final membrane preparation was stored at −20 °C.

Glycophorin from the carp red blood cell membranes was extracted using the lithium 3,5-di-iodosalicylate (LIS)-phenol method and streptomycin treatment [1]. After dialysis, the supernatant was stored at −20 °C as the glycophorin preparation.

### 2.3. Preparation of the Carp Glycophorin Carbohydrate Fraction

The carbohydrate fraction was prepared by β-elimination according to the method reported by Carlson [12]. The carp glycophorin fraction was incubated in 1.0 M NaBH_4_ and 0.1 M NaOH in the dark at 37 °C for 48 h under N_2_ gas. The reaction was neutralized by careful addition of 1 M acetic acid at 0 °C. The mixture was centrifuged at 2500× *g* for 30 min, and the supernatant was evaporated. The concentrate was dissolved in 5 mL of water and evaporated. This procedure was repeated three times. The preparation was washed with water and evaporated to form a syrup that was washed with 5 mL methanol followed by 5 mL ethanol. The concentrated oligosaccharide alditol preparation, which contained the carbohydrate fraction, was dissolved in 5 mL water. 

### 2.4. High-Performance Liquid Chromatography (HPLC)

The carbohydrate fractions were eluted using a Glyco-Pak DEAE column (Waters) [1]. The mobile phase consisted of 10 mM Tris-HCl (pH 7.6). The column was eluted with a continuous linear gradient of 0–100 mM NaCl. The flow-rate was 1.0 mL/min. A sample volume of 100 µL was injected onto the column during each run. The UV detector was set at 205 nm. The peak fractions (P-1 and P-2) were pooled. The HPLC procedure was repeated to increase the volume of each fraction. The oligosaccharide fractions (P-1 and P-2) were freeze-dried.

### 2.5. Desalting of Oligosaccharide Fractions

The oligosaccharide fractions were desalted using a GL-Pak Carbograph cartridge (500 mg/6 mL) according to our previous study [1]. To remove the contaminated acetic acid, the fraction was washed successively with water, 40% acetonitrile, 75% acetonitrile and 50% methanol. The preparation was evaporated and retained for analysis by GC-MS and NMR.

### 2.6. Thin-Layer Chromatography (TLC)

Thin-layer chromatography was performed to establish the purity of the oligosaccharides prior to extensive structural analysis. The oligosaccharide fractions (P-1 and P-2) were separated on a TLC plastic sheet silica gel 60 (Merck & Co., Inc., Darmstadt, Germany) and developed with a solution of *n*-propanol, 25% ammonia and water (6.2:1:4.2, v/v/v). The developed saccharides were visualized by spraying the plate with a diphenylamine-aniline-phosphate reagent [13].

### 2.7. Neutral Sugar Analysis

The oligosaccharide fractions (P-1 and P-2) were hydrolysed by 2.5 M trifluoroacetic acid at 100 °C for 6 h. After hydrolysis of the oligosaccharide fractions, the preparation was evaporated and dissolved in water. The preparation was applied to two columns (1.4 cm × 2.0 cm each) composed of Dowex 50 × 8 (H^+^ form) and 1 × 8 (CO_3_^2−^ form). The columns were washed with five bed volumes of water. The eluate was concentrated to obtain the neutral sugar preparation. For the determination of neutral sugars in each oligosaccharide fraction, we performed HPLC using a DX-500 and an ED 40 electrochemical detector with a gold working electrode and a Carbo Pac PA10 column (Dionex Co., Sunnyvale, CA, USA). The mobile phase consisted of 5 mM NaOH at a flow rate of 1.0 mL/min. The anhydro-Gal standard was obtained from Sigma Chemical Co.

### 2.8. NMR and GC-MS

Prior to the structural NMR analysis of the P-1 fraction, we prepared an asialo P-1 fraction in addition to the intact P-1 preparation. Sialic acid was released from the P-1 fraction by adding 5 mM HCl at 80 °C for 50 min under N_2_ gas. The reaction was terminated by adjusting the pH to 7.5 at 0 °C followed by desalting. ^1^H- and ^13^C-NMR and 2D NMR experiments (^1^H-^13^C HSQC, COSY, H2BC, HMBC, TOCSY and ROESY) were performed using JNM-α500 or JNM-ECA920 spectrometers (JEOL Ltd., Tokyo, Japan). The oligosaccharide preparation was permethylated following the method of Ciucanu and Kerek [14] prior to GC-MS analysis. Hydrolysis of the permethylated oligosaccharide was conducted with 2 M trifluoroacetic acid at 121 °C for 1 h*.* Reduction was performed by adding DMSO-NaBD_4_ at 40 °C for 1.5 h, and peracetylation was performed by adding acetic acid, 1-methyl imidazole and acetic anhydride successively. The GC-MS system consisted of a HP5890 gas chromatograph (Hewlett-Packard Co., Palo Alto, PA, USA), a JMS DX-303 mass spectrograph and a JMA DA5000 data module (JEOL).

## 3. Results

### 3.1. Homogeneity of P-1 and P-2 Fractions

**Figure 1:**
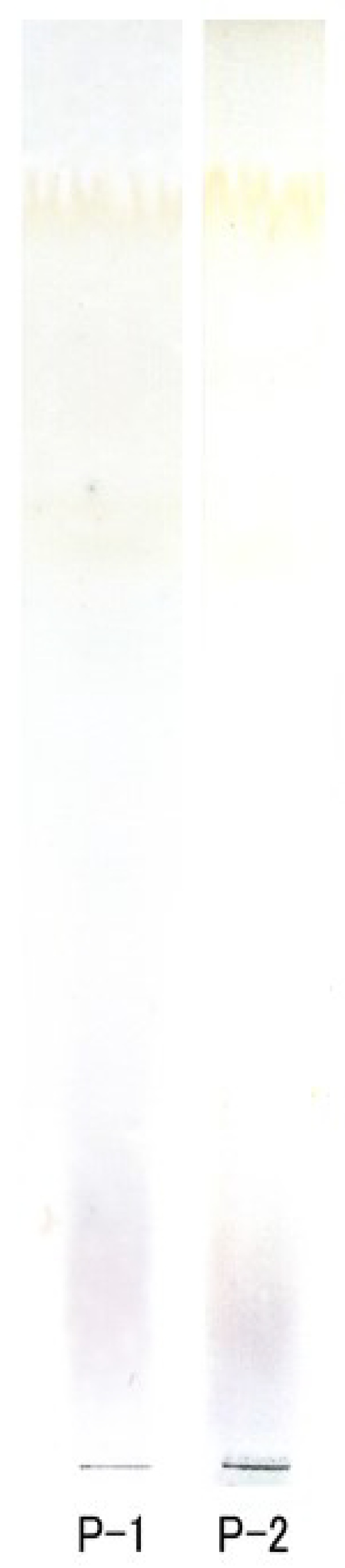
Thin-layer chromatogram of P-1 and P-2 fractions. Approximately 10 µg of oligosaccharide were applied to TLC.

Each oligosaccharide fraction from the last preparation step exhibited a single spot on the TLC sheet (Figure 1). After the washing step removed the contaminated acetic acid, the monosaccharide was not observed on the chromatogram.

### 3.2. Neutral Sugar Composition

**Figure 2:**
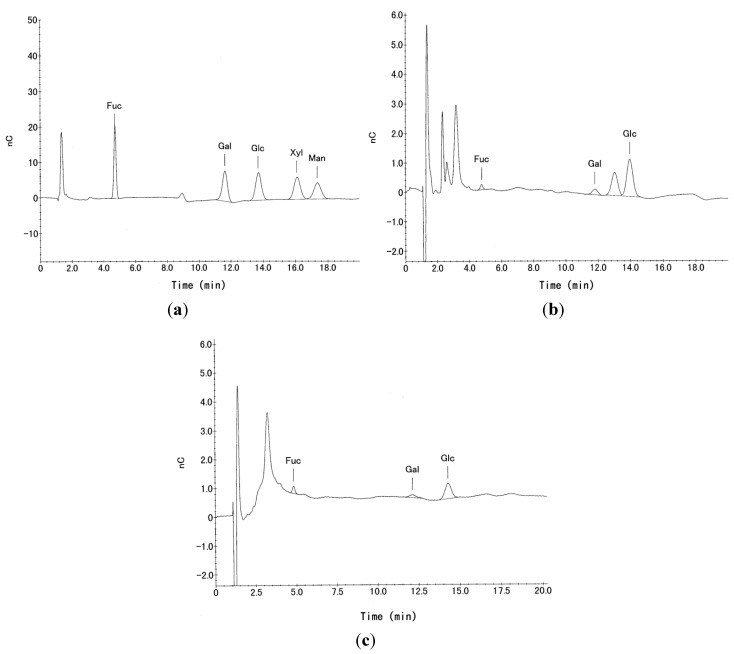
Chromatograms of (**a**) 0.1 ppm hexose standard and 100 µL injections of (**b**) P-1 and (**c**) P-2 hydrolysates from the HPLC electrochemical detector.

The neutral sugar composition of the oligosaccharide fractions (P-1, P-2) was detected using electrochemical detection after separation by HPLC. The peaks corresponding to Glc, Gal and Fuc were determined as neutral sugars in each fraction (Figure 2). The peak between Gal and Glc corresponds to the anhydro-Gal formed during hydrolysis. The unlabeled peaks at approximately 3 min were identified as contaminating salts in the oligosaccharide fractions.

### 3.3. NMR and GC-MS

**Figure 3:**
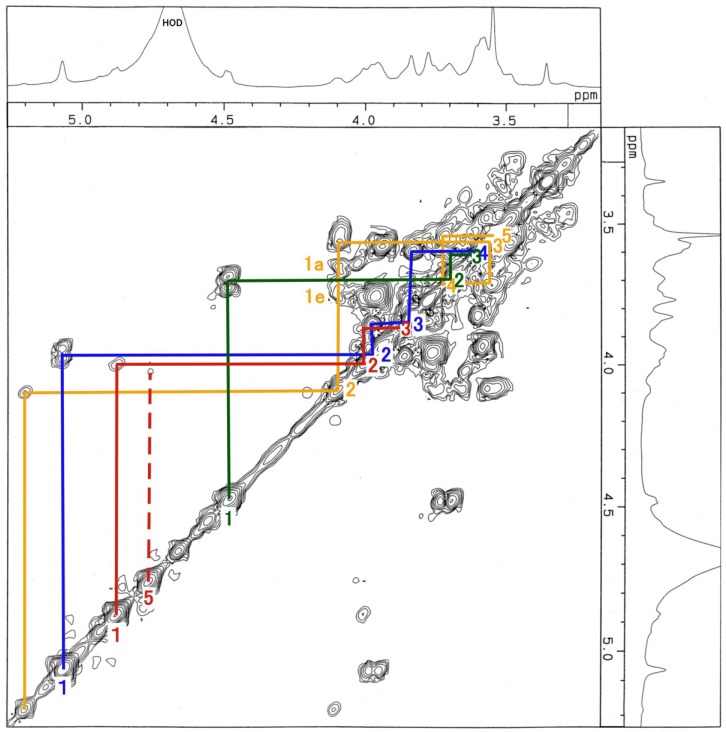
COSY spectrum of the asialo P-1 fraction. The blue line denotes the Glc residue; red line, Fuc; green line, Gal; brown line, GalNAc-ol. The number denotes the position of the proton. The spectrum was recorded for 256 scans at 500 MHz at 22.5 °C.

**Figure 4:**
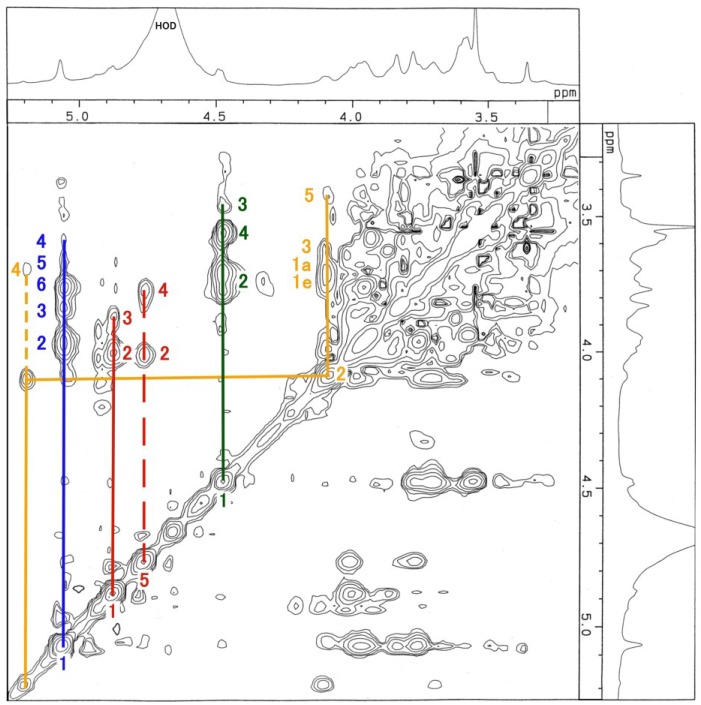
TOCSY spectrum of the asialo P-1 fraction. The blue line denotes the Glc residue; red line, Fuc; green line, Gal; brown line, GalNAc-ol. The number denotes the position of the proton. The spectrum was recorded in 64 scans at 500 MHz at 22.9 °C.

**Figure 5:**
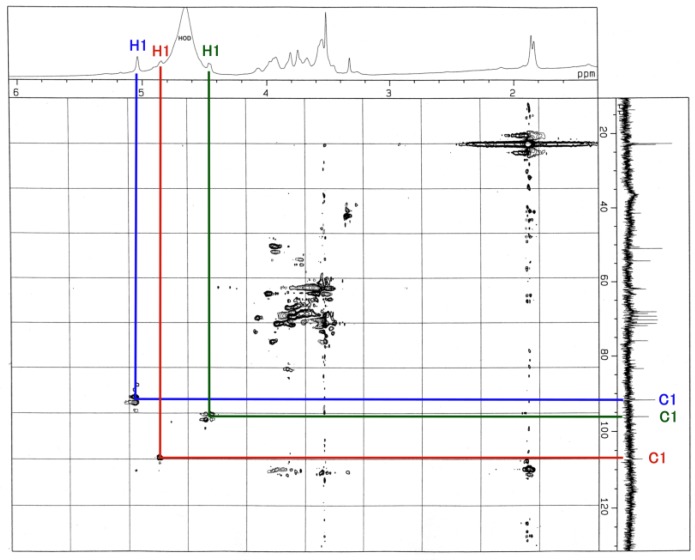
HSQC spectrum of the asialo P-1 fraction. The blue line denotes the Glc residue; red line, Fuc; green line, Gal. The number denotes the position of the proton and the carbon. The spectrum was recorded for 128 scans at 500 MHz at 22.3 °C.

**Figure 6:**
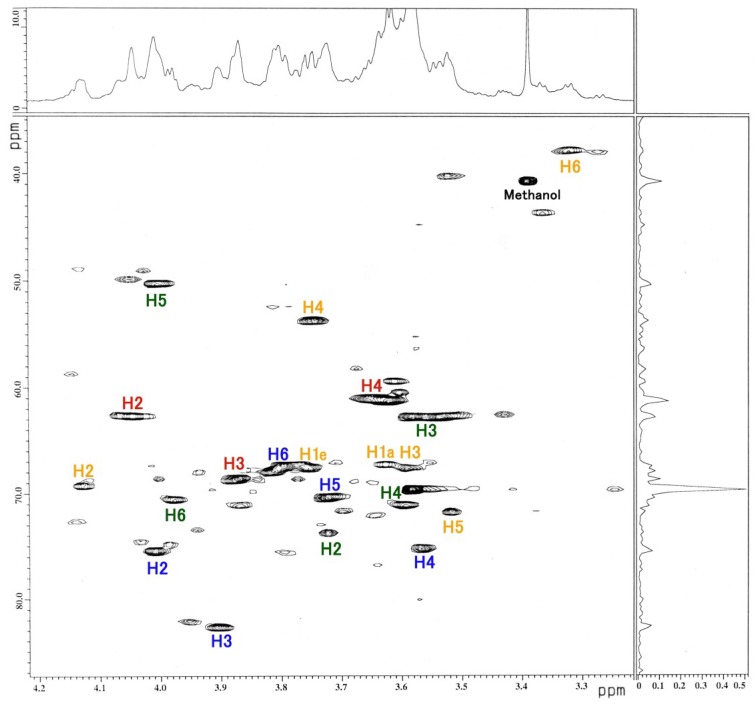
HSQC spectrum of the asialo P-1 fraction. The number denotes the position of the proton. The blue number denotes the Glc residue; red number, Fuc; green number, Gal.; brown number, GalNAc-ol. The spectrum was recorded for 64 scans at 920 MHz at 25.0 °C.

**Figure 7:**
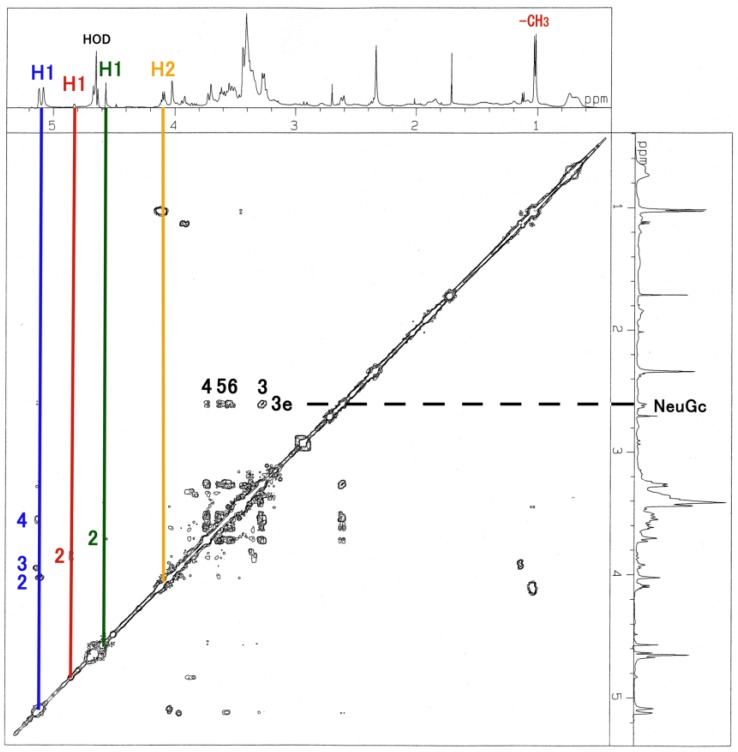
TOCSY spectrum of the intact P-1 fraction. The blue line denotes the Glc residue; red line, Fuc; green line, Gal; brown line, GalNAc-ol. The number denotes the position of the proton. The spectrum was recorded for 16 scans at 500 MHz at 20.3 °C.

The ^1^H-NMR, COSY (Figure 3), TOCSY (Figure 4), HSQC (Figure 5 and Figure 6) and H2BC spectra of the asialo P-1 fraction were obtained using 500- and 920-MHz spectrometers. The TOCSY and HSQC spectra revealed that the asialo P-1 fraction contained Glc, Fuc, Gal and GalNAc-ol in a molar ratio of 1:1:1:1 (Figure 4 and Figure 5). Based on the ^1^H-NMR spectrum, the H-1 signal of the Gal residue (δ = 4.463 ppm) exhibited a large coupling constant (*J*_1, 2_ = 8.0 Hz), which indicates a β-coupling to Gal. The remaining H-1 signals of αGlc, αFuc and the acetyl group of GalNAc-ol were at δ = 5.038, 4.846 and 5.174 ppm, respectively. The proton signal proportions on the TOCSY spectrum revealed an overall downfield shift in the resonance of αGlc and αFuc, except for the H-1 signals. All of the protons of the asialo P-1 fraction were characterized by TOCSY, COSY, HSQC and H2BC spectra (Table 1). In the TOCSY spectrum from the intact P-1 fraction, the H-3e signal of NeuGc (δ = 2.651 ppm) was detected (Figure 7). However, the H-3a signal was not observed. These results suggest that the structure of P-1 is not a chain, and two hexoses and one hexosamine are attached to a Gal residue.

**Table 1:** Chemical shifts of the asialo P-1 fraction.

Residue	Reporter Site	^1^H	^13^C
**GalNAc-ol**	1a 1e	3.626 3.763	67.34 67.34
	2	4.130	69.21
	3	3.592	67.58
	4	3.749	53.66
	5	3.519	71.71
	6	3.326	37.82
**αGlc**	1	5.103	91.01
	2	4.012	75.42
	3	3.905	82.62
	4	3.567	75.20
	5	3.727	70.37
	6	3.803	67.32
**αFuc**	1	4.917	106.5
	2	4.048	62.64
	3	3.875	68.61
	4	3.678	61.08
	5	4.764	71.60
	-CH_3_	1.234	20.10
**βGal**	1	4.518	95.39
	2	3.726	73.70
	3	3.583	62.76
	4	3.583	69.60
	5	4.007	50.27
	6	4.023	70.04

**Figure 8:**
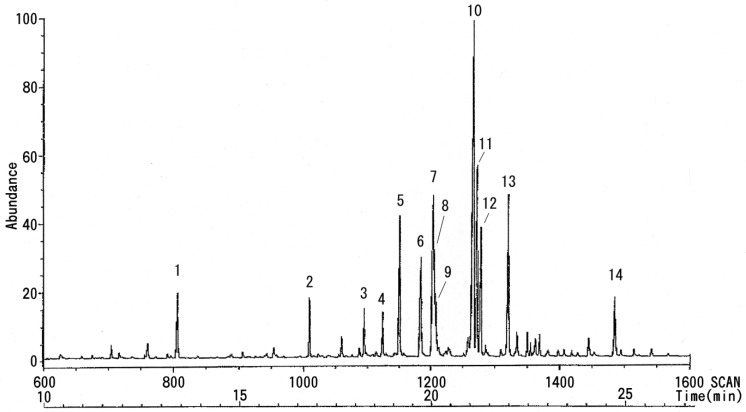
Total-ion chromatogram of permethylated oligosaccharides obtained from the P-1 fraction. The following peaks were identified: 2, 2,3,5-tri-*O*-methylpentose; 3, 3,5-di-*O*-methylpentose; 4, 6-deoxy-2,3,4-tri-*O*-methylhexose; 5, 2,3-di-*O*-methylpentose; 6, 2,3,4,6-tetra-*O*-methylhexose; 7, 2,3,4,6-tetra-*O*-methylhexose; 8, 2,3,4-tri-*O*-methylhexose; 9, 2,3,4-tri-*O*-methylhexose; 10, 2,3,6-tri-*O*-methylhexose; 11, 2,3,6-tri-*O*-methylhexose; 12, 2,4,6-tri-*O*-methylhexose; 13, 2,6-di-*O*-methylhexose; 14, 3,6-di-*O*-methyl-2-*N*-methylacetoamidehexosamine.

**Chart 1:**
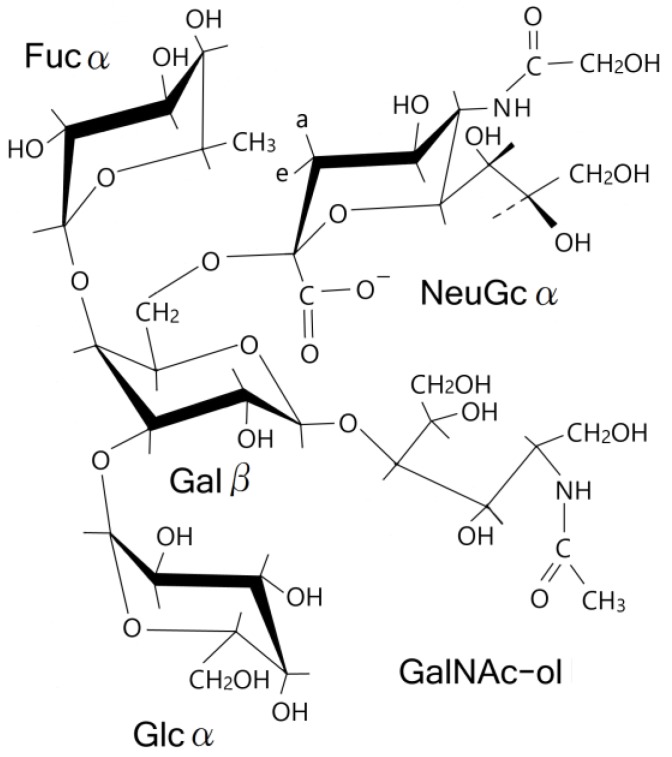
Assumed structure of P-1 oligosaccharide.

**Figure 9:**
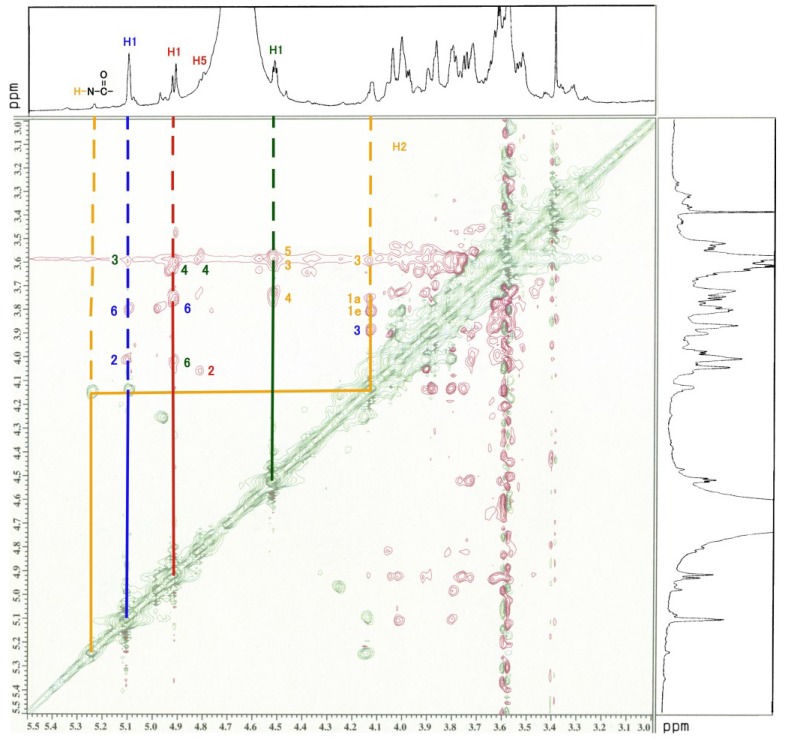
ROESY spectrum of the asialo P-1 fraction. The red signals denote the ROESY spectrum; green signals, COSY spectrum. The blue line denotes the Glc residue; red line, Fuc; green line, Gal; brown line, GalNAc-ol. The number denotes the position of the proton. The spectrum was recorded for 128 scans at 920 MHz at 25.0 °C.

Based on the GC chromatogram of the permethylated P-1 fraction (Figure 8) and the structure elucidation of each methylhexose fraction by MS spectra (Table 2), we determined that the structure of P-1 was NeuGcα2→6(Fucα1→4)(Glcα1→3)Galβ1→4GalNAc-ol (Chart 1). These determined glycosidic linkages were also suggested by the observation of ROESY correlation peaks (Figure 9).

**Table 2:** Structure elucidation on total ion chromatogram of permethylated oligosaccharides obtained from the P-1 fraction.

Peak No.	Linkage Site	GC Retention Time (min)	Peak Area	MS Retention Time (min)
1	−	15.297	13,928	13.23
2	Pen 1 →	18.747	11,924	16.47
3	→ 2 Pen 1 →	20.200	7,818	18.13
4	Fuc 1 →	20.747	13,925	18.42
5	→ 5 Pen 1 →	21.170	30,312	19.09
6	Hex 1 →	21.755	26,944	19.43
7	Hex 1 →	22.071	41,924	20.02
8	→ 6 Hex 1 →	22.153	28,993	20.04
9	→ 6 Hex 1 →	22.205	8,515	20.07
10	→ 4 Hex 1 →	23.162	91,934	21.05
11	→ 4 Hex 1 →	23.257	39,668	21.10
12	→ 3 Hex 1 →	23.361	25,210	21.17
13	→ 3,4 Hex 1 →	24.083	39,414	21.59
14	→ 4 HexNAc 1 →	27.002	11,999	24.43

Notes: Pen, pentose; Hex, hexose; HexNAc, *N*-acetylhexose.

## 4. Discussion

Glycophorins contain O-linked sialo-oligosaccharides, and the structure of these oligosaccharides from human red blood cell membranes has been analyzed [15]. The most commonly elucidated glycophorin oligosaccharides include the tetrasaccharide core NeuAcα2→3Galβ1→3(NeuAcα2→6) GalNAc-ol and the trisaccharides Galβ1→3(NeuAcα2→6)GalNAc-ol or NeuAcα2→3Galβ1→3GalNAc-ol [16]. A NeuGC-containing O-linked oligosaccharide has also been reported from horse, pig, goat and rabbit glycophorins, and the most commonly reported structure is a trisaccharide (Galβ1→3(NeuGcα2→6)GalNAc-ol). Other derivatives are synthesized by attaching NeuGc and Gal residues to the trisaccharide core to form a chain-like structure [16].

The carbohydrate fraction of carp glycophorin contained at least two types of O-linked oligosaccharides (P-1, P-2). The results of several sugar analyses suggested that the P-1 and P-2 fractions were composed of Glc, Fuc, Gal, GalNAc-ol and NeuGc.

Glc residue was not detected in the reported O-linked oligosaccharides from mammalian [16] and chicken glycophorins [7]. Guérardel *et al.* reported that *O*-glycans synthesized by nematodes contained the Glc residue [17], whereas the Fuc residue was detected in the O-linked oligosaccharides of human glycophorin A [18].

From the NMR spectra, the characterized proton signals of the asialo P-1 fraction revealed an overall downfield shift in the resonance of αGlc and αFuc, except for the H-1 signals. This O-linked oligosaccharide indicates a non-chain-like structure [16].

Furthermore, the linkage between Gal and GalNAc-ol is 1→4, unlike the 1→3 standard linkage for O-linked oligosaccharides.

The theoretical oligosaccharide composition (hexose:hexosamine = 3:1) derived from the NMR data differs from the experimental data in our previous study (hexose:hexosamine = 70:1) [1]. We hypothesize that the difference is due to the underestimation of hexosamine in the assay system. This may be caused by the 1→4 linkage of *N*-acetylhexosamine, which is known to result in low chromogen recovery [19].

The low reactivity of *N*-acetylhexosamine is caused by the difficulty of hexosamine delivery detection after permethylation. On the TOCSY spectrum from the intact P-1 fraction, the contaminated acetic acid peak was detected at δ = 2.383 ppm (Figure 7). It was necessary to remove the acetic acid from the desalted P-1 preparation for GC-MS analysis. After the successive washing process, the hexosamine delivery appeared on the GC chromatogram (Figure 8). Compared to the obtained GC chromatogram without the washing process (datum not shown), three permethylated pentose structures also appeared in the chromatogram. We assumed that these peaks originated from contaminated xylose fibers from the laboratory environment during the washing process.

The 1→4 linkage of *N*-acetylgalactosamine is unique compared with other O-linked oligosaccharides of mammalian origin. Interestingly, the β1→3 glycosidic linkage of xylan, which is a component of the seaweed cell wall, is unlike the standard β1→4 linkage of land plants [20]. It is possible to detect the β1→4 linkage of *N*-acetylgalactosamine in marine organisms.

## Abbreviations

NeuGc: *N*-glycolylneuraminic acid
Ac: acetyl
Fuc: fucose
Glc: glucose
Gal: galactose
GalNAc-ol: *N*-acetylgalactosaminitol
